# Massive Vaginal Hemorrhage after Cesarean Delivery: A Newly Recognized Mechanism of Postpartum Hemorrhage due to Lateral Uterine Artery Injury

**DOI:** 10.31662/jmaj.2025-0531

**Published:** 2026-02-06

**Authors:** Yutaka Iwagoi, Munekage Yamaguchi, Kana Hirao, Jun Sakata, Yasuhiro Yamamoto, Akihito Sagara, Saori Yoshimura, Fumitaka Saito, Takeshi Motohara, Eiji Kondoh

**Affiliations:** 1 Department of Obstetrics and Gynecology, Faculty of Life Sciences, Kumamoto University, Japan

**Keywords:** postpartum hemorrhage, cesarean delivery, uterine artery injury, dynamic computed tomography, vaginal bleeding, labor arrest

## Background

Postpartum hemorrhage (PPH) remains a leading cause of maternal mortality worldwide, accounting for approximately 8% of maternal deaths even in developed countries ^(1)^. Identification of the bleeding source is crucial for achieving effective hemostasis. Traditionally, dynamic computed tomography (CT) has not been routinely used for PPH. However, its use is increasing for evaluating treatment-resistant cases and for guiding optimal hemostatic management ^(2), (3), (4), (5)^. Accordingly, dynamic CT is reshaping current understanding of PPH mechanisms. Recently, a newly recognized mechanism of massive vaginal hemorrhage following cesarean delivery has emerged. Here, we report a series of cases demonstrating intrauterine extravasation from lateral uterine artery injury―a mechanism distinct from conventional retroperitoneal bleeding.

## Hemorrhage after Cesarean Delivery

Cesarean delivery accounts for 25%-40% of severe PPH ^(5), (6)^. Historically, most of these treatment-resistant uterine hemorrhages were attributed to intrauterine bleeding sources, such as underlying placenta accreta spectrum. During normal pregnancy, 100-150 uterine arteries open into the endometrial cavity ^(7)^, and following placental separation, myometrial contraction compresses these vessels to achieve physiologic hemostasis. However, recent reports have described cases of refractory PPH in which a single, focal arterial channel continues to bleed into the uterine cavity ^(2), (3), (4), (5)^. The mechanism is not fully understood, but impaired decidualization may be a factor, as placenta accreta spectrum is known to be more common in women after assisted reproductive technology using hormone replacement cycles ^(8)^. Clinically, such cases are characterized by pinpoint bleeding rather than diffuse oozing from the placental bed ^(2), (3), (4), (5)^. Management strategies, therefore, should move beyond traditional compression sutures or hysterectomy toward targeted uterine artery embolization or precise surgical hemostasis when laparotomy is required ^(9)^.

## Case Observation and Findings

The expected course of uterine artery injury after cesarean delivery involves retroperitoneal bleeding that subsequently extends into the abdominal cavity (Video 1). In contrast, dynamic CT has revealed cases in which disruption of the uterine artery along the lateral uterine wall resulted in extravasation into the uterine cavity, presenting clinically with massive vaginal bleeding (Video 2). We retrospectively reviewed patients transferred to our institution for critical PPH between January 2023 and December 2024. Among 24 cases of PPH after cesarean delivery, six (25%) patients demonstrated contrast extravasation on dynamic CT originating from injured sites of the uterine artery running laterally along the uterus. Two followed the conventional retroperitoneal pattern, requiring laparotomic hemostasis. Remarkably, four cases (17%) showed intrauterine extravasation from the uterine artery adjacent to the lateral uterine wall, all of which were successfully managed with uterine artery embolization. Notably, these four patients had undergone cesarean delivery for arrest of labor after full or near-full cervical dilation.

To further evaluate the broader relevance of this finding, we conducted a supplementary review of dynamic CT images from 180 PPH cases collected in a nationwide cohort at 43 tertiary centers in 2021 ^(5)^. Two additional patients demonstrated the same atypical pattern of intrauterine extravasation attributable to lateral uterine artery injury; both had also undergone cesarean delivery for arrest of labor. To our knowledge, this newly recognized mechanism―lateral uterine artery injury leading to intrauterine hemorrhage manifesting as catastrophic vaginal bleeding―has not been previously described. Without dynamic CT, such cases might have been misclassified as unexplained hemorrhage or even as uterine-type amniotic fluid embolism.

Given that this was a retrospective review of medical records, individual informed consent was waived, and an opt-out procedure was implemented in accordance with institutional guidelines.

## Clinical Implications

In cesarean deliveries for labor arrest, manual elevation of the deeply engaged fetal head acutely stretches the already thinned lower uterine segment. This maneuver may extend the hysterotomy site laterally or posteriorly and precipitate disruption of the ascending or descending branches of the lateral uterine artery. These arterial branches run along the lateral uterine wall and lie exceptionally close to the cervico-isthmic region when markedly thinned at full dilation. Since the lower uterine segment is paper-thin under such conditions, subsequent hysterotomy closure may potentially leave the vascular breach opening directly into the uterine cavity. As a result, arterial bleeding manifests as sudden, profuse vaginal hemorrhage rather than intra-abdominal bleeding.

This mechanism underscores the need for heightened vigilance in cesarean deliveries for labor arrest, where thinning of the cervico-isthmic region is common. Clinically, such cases can be misleading: despite catastrophic external bleeding, intra-abdominal blood loss may be minimal, potentially delaying recognition of the true vascular source. Dynamic CT plays a pivotal role in distinguishing this atypical mechanism of PPH. When CT reveals a focal arterial extravasation, uterine artery embolization may offer rapid and definitive hemostasis. Although ultrasonography is widely available, transabdominal Doppler imaging is unlikely to detect a focal arterial jet because the bleeding point typically lies deep near the internal cervical os and is generally not suitable for definitive assessment in this setting. Most importantly, these observations call for increased attention to surgical technique in labor arrest cases, particularly with respect to minimizing lateral vascular injury and thoroughly inspecting the hysterotomy site before closure.

## Future Directions

Although uncommon, this newly recognized mechanism may account for a non-negligible proportion of critical PPH after cesarean delivery. Awareness of this entity, particularly in cases of labor arrest, may enhance intraoperative vigilance, promote early diagnostic use of dynamic CT, and facilitate timely interventional management. Broader recognition may ultimately contribute to reducing maternal morbidity from PPH worldwide. Finally, multicenter collaborations are warranted to define the incidence, clarify risk factors, and refine management strategies for this newly recognized clinical entity.

## Article Information

### Author Contributions

Yutaka Iwagoi and Eiji Kondoh identified the new clinical concept. Yutaka Iwagoi, Eiji Kondoh, and Kana Hirao reviewed the computed tomography images. Munekage Yamaguchi, Akihito Sagara, and Jun Sakata collected imaging data. Yutaka Iwagoi, Kana Hirao, Yasuhiro Yamamoto, Jun Sakata, Saori Yoshimura, Fumitaka Saito, and Takeshi Motohara provided clinical management. Yutaka Iwagoi and Eiji Kondoh drafted the manuscript, and all authors revised it critically and approved the final version.

### Conflicts of Interest

None

### IRB Approval Code and Name of the Institution

Approved by the Ethics Committee of Kumamoto University Hospital with an opt-out consent procedure (No. 3366).

## Supplement

Supplementary Material Video 1. Typical postpartum hemorrhage after cesarean delivery.
Dynamic CT images (1-mm slices, sagittal, axial, and coronal) are shown, with the axial plane scrolling through consecutive slices to illustrate uterine artery injury leading to retroperitoneal and intraperitoneal bleeding. The sagittal and coronal images are static and provide anatomical context.
CT: computed tomography.
Video 2. Atypical postpartum hemorrhage after cesarean delivery.
Dynamic CT images (1-mm slices, sagittal, axial, and coronal) from two patients demonstrate lateral uterine artery injury resulting in focal extravasation into the uterine cavity, clinically presenting with massive vaginal bleeding. In both cases, the axial plane scrolls through consecutive slices to visualize the hemorrhage, while sagittal and coronal views provide static anatomical context.
CT: computed tomography.
